# The contagious leader: a panel study on occupational stress transfer in a large Danish municipality

**DOI:** 10.1186/s12889-022-14179-5

**Published:** 2022-10-07

**Authors:** Lærke Bonnesen, Signe Pihl-Thingvad, Vera Winter

**Affiliations:** 1 Department of Political Science and Public Management, University of Southern Denmark, Odense, Denmark; 2 Schumpeter School of Business and Economics, University of Wuppertal, Wuppertal, Germany

**Keywords:** Stress contagion, Stress spill-over, Public management, Psychosocial work environment, Occupational stress, Manager stress, Employee stress, Well-being, Panel study

## Abstract

Occupational stress has immense economic and health-related consequences for individuals, organizations, and societies. In this context, the question of whether and how stress among managers transmits to their subordinates is highly interesting, yet not profoundly researched. This study aims to empirically investigate the effect of manager occupational stress on the development of subordinate stress and for how long such effects last in time. We exploit a unique panel dataset based on three different surveys among employees from a large Danish municipality, covering 5,688 employees and their 473 immediate managers between 2016 and 2020. We analyze this data using a fixed effects estimator with clustered robust standard errors, allowing us to significantly reduce potential endogeneity issues. The study shows that managers do in fact ‘transmit’ stress onto their employees, that the relationship is detectable a full year after the initial transmission of stress occurred, and that such an effect fades within additional two years. Our study serves to emphasize the great importance of the psychosocial wellbeing of managers as ‘nerve centers’ for entire job teams and urges organizations to treat stress among personnel on management levels with a high degree of concern.

**Supplementary Information:**

The online version contains supplementary material available at 10.1186/s12889-022-14179-5.

## Introduction and theoretical considerations

During the past three decades, occupational stress has increasingly been identified as a public health concern with severe ramifications for individual well-being and organizational performance. Perceived occupational stress – defined as an individual experience of being unable to cope with the demands of the work environment [1] – is the second-most reported health problem among European workers [2], as well as a major cause of work absenteeism [3, 4], low organizational commitment [5], inferior job performance [6], and poor mental and physical health [7]. In Denmark, the prevalence of perceived stress among working individuals saw an increase from 21 to 25% from 2010 to 2017 [4] and has been shown to affect both short- and long-term sickness absence [8].

Manager behavior is generally recognized as a very important determinant of subordinate well-being at work [6, 9, 10]. A multitude of studies point to the significance of leadership in fostering a healthy work environment [11–15]. Few studies have, however, paid specific attention to the effects of manager stress on the well-being of subordinate workers [16]. Though the affective state of managers is clearly a very important dimension of leadership, there is an apparent lack of research interest in manager affect [9], including stress among managers. This is all the more surprising knowing that stress is a prevalent health issue among managers [3], and that stress limits an individual’s ability to plan ahead, make rational decisions, self-regulate emotions (e.g. aggravating the tendency towards aggression), and provide social support [6] – all abilities that are commonly expected of managers striving to secure a healthy work environment for their employees.

Leaders can transmit stress to employees through different pathways which can be categorized broadly as *direct* and *indirect* transmission mechanisms. The direct pathways work through the interaction between leader and employee. From studies in social psychology, convincing evidence that emotions are highly ‘infectious’ and can spill over between parties in a social relationship through interaction and communication has accumulated during the past three decades [17]. This is especially true in relationships characterized by high interdependence [18] and inequality in power [19], such as manager-subordinate dyads. Defined as the transfer of moods and emotions from one person to another, emotional contagion represents a comprehensive academic field within social psychology. Well-recognized scholars like Hatfield, Cacioppo & Rapson (1993) and Elfenbein (2014) have broadened our understanding of how moods and emotions are not merely individual states but can (given different circumstances) easily be transferred between individuals [20, 21]. Through mimicry of facial expressions, tone of voice etc., managers can transfer moods and emotions onto their employees through ‘primitive’ mood contagion, which refers to the automatic – and mostly unconscious – process of emotional convergence among interaction partners (due to manager’s emotional displays) [17, 20]. Moreover, moods and emotions can also be transferred directly in more conscious ways, such as through social comparison and role-modeling, which occur when employees more or less actively align their own reactions, emotions and moods according to those of their leader [21–23]. In line with the Emotions as Social Information Model [24], the emotional displays of a manager are used as social cues for contextually ‘appropriate’ emotions and social reactions by their subordinates.

To be aware of such emotional spill-over effects between leaders and employees is especially important in a public sector context. Several studies have shown that public sector employees generally have more pronounced pro-social values [25]. This pro-social orientation implies that employees empathize to a greater extent with the other party in relationships at work (both with clients and co-workers). In situations where employees show empathy towards a stressed leader, there is a high risk of facilitating the transfer of emotions, as empathy evokes an emotional response to and often mirroring of the other’s feelings [21, 26]. Furthermore, the majority of employees in local governments (as is the case in this paper) are employed in service delivery which is often intense in emotional labor. The employees are therefore used to participating in such emotional processes expressing empathy [27]. These particular aspects of public service delivery imply that one should expect ‘empathetic’ stress transfer to be especially common among public employees. In sum, public managers can affect the emotional state of their subordinates through direct pathways in more or less deliberate ways through daily interaction and communication.

Beyond the direct transfer of emotions, stress can be transferred indirectly by means of environmental and behavioral changes brought about in the wake of leader stress. As Conservation of Resources Theory states, changes in the environment constitute a pathway that influences employee well-being [28]. For instance, changes in the leadership style of managers who, in response to stress, may withdraw and ‘conserve resources’ [28] and fail to plan and organize the work sufficiently and take responsibility can cause stressful changes to the employees’ work environment and thereby transfer stress to the employees indirectly. Simultaneously, stressed leaders can become less prone to displaying positive leadership behaviors (e.g. providing less social and instrumental support) and become more prone to displaying harmful ones (such as abusive behaviors) [6, 16, 29]. According to Affective Events Theory, workplace events, such as changes in leadership behavior, influence employees’ affective emotions [30] and trigger either a positive or negative appraisal in employees, depending on the nature of the change. Thus, changes in leadership behavior due to stress, as outlined above, can lead to stressed employees by route of negative appraisal. Overall, we therefore expect that increases in perceived stress among managers increases perceived stress among their employees.

The few studies that have empirically investigated the relationship between manager and subordinate stress rather consistently support this phenomenon of ‘stress contagion’ [26, 31–36]. However, these studies are often based on time-invariant data (limiting the potential for causal inference) and/or data from a sports-setting (the relevance of which may not travel well to a public sector work context). In addition, no studies have, to the best of our knowledge, considered the length of stress contagion effects once first induced. In the existing literature, the longest timespan examined is an interval of three month in two studies by Chullen (2014) [26, 31]. Thus, the question of how long a stress contagion effect takes hold has not been investigated so far, and data collected within a longer timespan (for example intervals of one or more years) have never been examined. This is, however, both theoretically and empirically important. Even a relatively small effect size may have rather significant real-life health and performance consequences for individuals and the organizations they belong to if that effect is long-lasting. Given the limitations of the existing pool of studies with no theoretical considerations of the possible timespans, only very few repeated-measure analyses and, among these, a rather short-termed timespan in measurements, there are neither strong theoretical nor empirical evidence on the length of a possible contagion effect. We therefore use an exploratory approach in this paper to investigate the possible timespan (here, intervals of one and three years, respectively) of the contagion effect.

Thus, in this study, we aim to empirically investigate (1) the effect of perceived manager stress on the development of subordinate stress in a public sector context, and (2) how long such effects may last in time. We attempt to accommodate the shortcomings in the existing literature on stress ‘spill-over’ effects by analyzing unique panel data from a sample of 5,688 employees and their 473 immediate supervisors in a large Danish municipality and using temporal data with different time intervals to investigate the duration of stress contagion effects.

## Methods and data

### Study design

To investigate stress contagion effects in manager-employee dyads, we utilize data from a large longitudinal cohort study of public employees, PUMA-WSC^1^ [37]. This study follows a total population of approximately 8,000 employees and their managers in Esbjerg Municipality in Denmark from 2016–2025 and focuses on employee well-being and manager behavior. The employees and managers in the study are all following the same regulations regarding personnel policies and management guidelines. They work in a broad range of professions within the municipality, though mainly within the fields of human services and administration. All data are collected by the municipality in collaboration with the research group on PUMA-WSC.

For the current study, we employ data from a survey on sickness absence antecedents from 2016 (response rate: 68%) as well as two surveys on psychosocial work environment from 2017 (response rate: 85%) and 2020 (response rate: 77%). While the surveys in 2017 and 2020 included the entire population of municipal employees in their sampling efforts, the survey from 2016 collected data on the basis of a stratified sample of employees with an oversampling of employees without a record of sickness absence. The oversampling of never-sick employees means that this subpopulation makes up 38.9% of the survey sample, while, in reality, they barely compose one-fourth (24.5%) of the actual population of employees in Esbjerg municipality. To minimize this potential source of bias, the sample has been corrected with the application of sampling weights (weights that denote the inverse of the probability that the observation is included) in the statistical analysis. Weighting the data in accordance with the population statistics has the benefit of making the sample and thus the analysis more representative of the population of interest.

The discontinuity in survey wave intervals (one year between the first and second, and three years between the second and third) provides a unique opportunity to study the effect of time on stress contagion. In the analysis, we limit our attention to 5,688 employees and their 473 immediate managers. Immediate managers are chosen as point of focus due to their close proximity to the day-to-day work life of the employees, in addition to serving as primarily responsible for the personnel management of these same employees. All observations include repeated measures to create a panel consisting of employees with no managerial responsibility enriched with information on each employee’s immediate supervisor. We combine data on managers’ self-reported stress with data on employees’ self-reported stress. Thereby we avoid common source bias and harvest the benefits of panel data.

For analyses with two points in time, balanced panels are necessary for performing a fixed effects analysis. In order to build the final panel dataset suitable to statistically analyze the matter of stress contagion in manager-employee dyads and the length of such effects, the sample has been restricted to (1) employees without management responsibilities; (2) who have been employed for a minimum of two consecutive time periods out of the researched three; (3) with available identification of respondents’ immediate managers and their perceived stress for the corresponding time periods; (4) and with stable manager-employee dyads (answering to the same manager) for at least two consecutive time periods. These necessary restrictions have naturally reduced the initial sample. Furthermore, the analyses containing 2016-data (Model I) is limited by the sampling strategy of the 2016-survey: whereas the 2017- and 2020-surveys were population-based, the 2016-dataset contains responses from a stratified random sample of 4,578 employees (equaling 48% of the employee population at the time). While the surveys have been conducted with a priority to high response rates by reserving time for questionnaire response during working hours for all employees, non-response and expected panel attrition, as well as partial survey responses, still reduce the N from an initial population of 8,301 municipal workers to a working dataset of 5,688 employees and their 473 immediate managers. This results in an overall dataset of approximately 69% of the employee population in Esbjerg municipality (for some years – especially 2016 – the number is lower, and for others – especially 2020 – higher) which we deem reasonable for analysis. Moreover, the dataset reflects the characteristics of the population to a reasonable degree: The sample has a majority of female employees (81.7%), which is characteristic in a public sector context [38]. The sample employees had on average 10.5 years of seniority in 2020, and the average age was 46.8 years in the same year.

### Measures

#### Perceived stress

Among managers and employees is measured with a four-item index taken from the Copenhagen Psychosocial Questionnaire (COPSOQII). The items include experiences of having trouble relaxing, irritability, tenseness, and feeling stressed during the past four weeks (Cronbach’s Alpha for employees = 0.86 (2016) / 0.88 (2017) / 0.88 (2020); Cronbach’s Alpha for managers = 0.87 (2016) / 0.89 (2017) / 0.87 (2020)). It is a measure of self-reported stress that is highly recognized within existing stress research, as it covers known cognitive and behavioral symptoms of stress. The measure of perceived stress has been validated several times (for example [39, 40]). Documentation and psychometric properties for the index can be found in Bjorner & Pejtersen (2010) [41]. The measure of stress is the same in all three surveys. However, the wording used in the 2016 survey diverges slightly from the wording of the 2017 and 2020 surveys with regard to the specified recall period, referring to *“the past six months”* instead of *“the past four weeks”.* As studies point to a ‘mental anchoring effect’, people tend to evaluate such questions with an inadvertent priority to their most recent memories [42, 43]. Therefore, and due to a very similar variable distribution across the recall periods, the stress variables are considered comparable across years. Employee stress (2017, 2020) is utilized as a dependent variable in the statistical analysis [index range after mean-centering: -1.11, -2.89], while supervisor stress (2016, 2017) is utilized as an independent variable [index range after mean-centering: -0.96, -2.79].

Due to the panel structure of our data, controlling for time-invariant variables such as gender and age is unnecessary, as such individual heterogeneity is implicitly controlled for by way of the statistical model. However, some additional control variables that represent circumstances which may affect both the dependent and independent variables and vary over time are included in the analyses:

#### Work environment

This indicator – available in all three of the surveys – is utilized as a control variable in the statistical analysis to ensure that changes in the work environment during the time periods do not cause a spurious correlation between manager and employee stress who, inter alia, share a similar environment. Psychosocial work environment has proven highly predictive of occupational stress in past studies [44]. The indicator for work environment contains employee’s evaluation of their work environment (degree of agreement with the statement, “all in all, there is a good work environment at my place of work”) on a 5-point Likert scale [index range after mean-centering: -2.79, -1.22], with higher values representing more positive appraisals. The item is taken from the Copenhagen Psychosocial Questionnaire (COPSOQ). It is a single item that has been validated as a global measure of employees’ perceptions of their work environment. It has been shown to correlate with other important dimensions of in the psychosocial work environment, including but not limited to work commitment, perception of job insecurity, work-life balance, and job satisfaction [39]. The inclusion of this item in the analyses ensures that any changes (e.g. higher work pressure or eroding social atmosphere) in the shared work environment of managers and employees do not cause omitted variable-bias in the estimates of the main explanatory variable.

#### Manager-employee relationship

Additionally, we control for the perceived quality of the dyadic relationship between manager and employee. The measure comprises four items of employee assessments of immediate managers’ ability to show consideration for the employees’ views and needs, solve tangible problems, and understand the work performed in the unit, as well as having a relationship with the manager characterized by mutual respect and appreciation (Cronbach’s Alpha = 0.91 (2017) / 0.91 (2020)). The scale has been adapted from a validated questionnaire on workplace social capital from the Danish National Research Centre for the Working Environment [45]. These items are all assessed on a five-point Likert scale, and the index has been transformed to reflect this scaling [index range after mean-centering: -2.03, -2.00]. However, these items were not included in the survey from 2016, and therefore Model I (2016–2017 analysis) does not contain this particular control.

### Statistical analyses

To analyze the panel dataset, a fixed effects estimator is employed. The robust standard errors of the model are adjusted for manager-clusters, as the 5,688 employees are embedded in 473 work units with corresponding personnel managers (average supervisor span-of-control: 12.5 subordinates). One of the main advantages of the fixed effects estimator is that, by design, the model accounts for all unobserved individual heterogeneity that remains constant across the time period, strengthening the statistical grounds for causal inference.

The statistical analyses are conducted in two steps: first, an analysis of the potential ‘contagion’ effect of manager stress on employee stress across a one-year period (Model I: 2016–2017); secondly, an analysis of ‘contagion effects’ across a three-year period (Model 2: 2017–2020). Even though both analyses, with their relatively wide time frames, represent conservative tests of the hypotheses as compared to the existing literature, the one-year time-gap between data collection in 2016 and 2017 involves a greater likelihood of uncovering any occurred process of stress contagion compared with the three-year time-gap between 2017 and 2020.

Postestimation diagnostics reveal no problems with influential outliers, non-linear correlations, or severe multicollinearity. A central assumption of regression analysis is that error terms are uncorrelated, and this assumption can be violated by the presence of autocorrelation in the data – however, the clustered data structure is handled in the statistical model, i.e. with fixed effects and cluster-robust standard errors. The robust estimation of the standard errors simultaneously mitigates any potential problems with heteroscedasticity.

As the work environment might also function as a mediator, i.e., manager stress might also affect employees’ work environment, we conducted additional analyses without controlling for the work environment variable (analyses have been run by way of stepwise estimation). Moreover, we have conducted sensitivity analyses with additional control variables (perceived cooperation in one’s team/workgroup, and the degree of employee identification with the organizational values of the workplace) in order to test the statistical stability of the results reported in this paper. These variables were only available for 2017 and 2020 and could therefore not be included in Model I. The results of Model II proved stable to the inclusion of different exogenous variables. Lastly, in order to check the robustness of results, further sensitivity analyses have also been carried out on the basis of a random effects estimator. In comparing the models, results from the Hausman Specification Test render support for the utilization of a fixed effects estimator for the present purposes.

## Results

In the following, results from the statistical analyses are presented, with results from the first two waves of data covering the one-year period of 2016–2017 (Model I) presented first, and results from an analysis of the last two waves of data covering a three-year period from 2017–2020 (Model II) second. The results are shown in Table 1 below.

**Table 1:** Statistical analyses

	**Model I:** *One year* (2016–2017)		**Model II:** *Three years* (2017–2020)	
	**Step 1**	**Step 2**	**Step 1**	**Step 2**
**Independent variable**				
Leader stress	.12 (.03)*	.10 (.04)*	04 (.04)	03 (.04)
**Control variables**				
Eval. of work environment	-	-.19 (.00)***	-	-.19 (.00)***
Supervisor-employee relationship	-	-	-	-.17 (.00)***
**Model parameters**				
N (# of unique obs.)	1,510	1,506	5,358	5,288
n (# of leader clusters)	262	262	452	452
Constant	-.02 (.00)***	-.01 (.03)*	.00 (.00)***	.02 (.00)***

Beta-coefficients of mean-centered predictors and standard errors in parentheses

*P*-values denoted as follows: α < .05*, α < .01**, α < .001***

The fixed effects analysis of the data from the years 2016 and 2017 spanning one year reveals a statistically significant correlation between manager stress and employee stress with the expected positive direction of the relationship: When managers report symptoms of stress, employees do as well, controlling for the work environment shared in the work unit. The beta coefficient of 0.10 indicates that, when managers’ stress increases by one unit on a five-point-scale, employees’ stress increases by 0.10 units. Put simply, it seems that 10 pct. of a manager’s stress increase may ‘spill-over’ into employee’s stress as long as one year after.

Looking to Model II, the fixed effects analysis of the data from the years 2017 and 2020 spanning three years reveals no significant correlation between stress symptoms among managers and corresponding symptoms among their employees. This is true even with a substantially larger sample size compared to Model I. It thus seems unlikely that the lack of a stress contagion effect can be explained with reference to the statistical power of the model. The lack of a significant beta-coefficient holds true both with and without the statistical controls for work environment and manager-employee relationship.

The statistical significance of the main term of Model I and the corresponding lack of statistical significance of the main term in Model II support the assumption that a stress contagion effect fades with the passing of time – in the present data after one and before three years after the initial incident of stress contagion.

Besides the stress variables of main interest, the control variables for work environment (in both models) and the manager-employee relationship (in Model II) significantly and negatively correlate with employee stress in both models.

## Discussion

Occupational stress is an increasing health-related problem. Studies have pointed out that manager stress may be central to employees’ experience of occupational stress over time. However, the few existing studies that employ panel data to investigate stress transfer in manager-employee dyads only estimate contagion effects with time-periods up to three months [26, 31, 34, 35], missing the opportunity to study the length of such effects.

In this study, we find that a stress contagion effect among managers and their employees can be traced a full year after the manager reports symptoms of occupational stress. Approximately 10 percent of the manager’s stress translates into employee stress. This is no small amount and is well in line with findings from other similar panel studies, such as Chullen (2014) who found that approximately 11 pct. of the variation in employee burnout could be explained by similar symptoms among managers three months before [31]. Even though the present study serves as a rather ‘conservative’ test, we show that there is a spill-over effect from managers to employees after a full one-year timespan. One year is certainly a long time to suffer from the effects of manager stress contagion and it may have severe consequences for employees. The long time-span of effects found in the present study is especially interesting if we compare them with results from studies that examine the average duration of severe stress reactions (reactions that are so severe that they induce long-term sickness absence): such studies show that it takes between 55 days [46] and 25 weeks [47] before employees are able to return to work after a stress-related sick leave – significantly less than a year. While the effect found in our analysis probably partially relies on the fact that manager stress might prevail after the initial measurement, it is reasonable to assume that the effect size of the stress contagion would probably be substantially larger if the follow-up measurement had been just one or two months after the initial measurement.

Furthermore, the empirical analysis in our study also points to a null effect of stress contagion three years after the initial reporting of stress symptoms among managers. As such, the study rather uniquely contributes to a tentative and new understanding of the longevity of stress contagion effects – something that past studies, with shorter timelines or a simple lack of time-variant data, have not been able to shed light on. Thus, it seems that the effect of manager-to-employee stress transfer declines to the point of statistical insignificance sometime after the first year has passed and before the passing of three years. This points to the validity of the first finding – which is indicative of a rather pervasive effect – and that any effect of supervisor stress on the emotional state of employees seems to have an ‘expiration date’ – all else being equal.

The results should be taken as additional caution to organizations that perceived stress at manager levels should be treated with a high degree of concern. As stress is the second-most widespread health problem among European workers [2], and occupational stress has proven extremely costly not only for the affected individuals but also for public and private organizations (in terms of absenteeism and reduced productivity), as well as society at large [48], even relatively limited increases in occupational stress can have severe implications. This emphasizes the importance of directing organizational attention to the development of occupational stress at manager levels. Though this point may seem self-evident, stress among managers is still a rather restricted research area [3]. This may well reflect the prevalence of a persistently conservative leadership ideal in which coping with stress may be taboo.

This study is unique in the sense that we employ a large sample with original, time-variant data on organizational members using a validated measure on perceived stress symptoms among both managers and employees. One limitation which, although it doesn’t invalidate the conclusions of this study, does ‘pixelate’ the findings somewhat, is the measure of time with one- and three-year time intervals. It would be interesting for future studies to follow the contagion effect on monthly or even weekly time intervals over a longer period to investigate the development and fading of stress contagion effects over time more closely. It may be that the development of such stress transfer processes is nonlinear, perhaps quadratic, in nature. Since the eventual ‘fading date’ of a stress contagion instance can have rather long prospects, we urge future studies to employ more fine-grained time data in order to study the development of stress contagion processes and their effects with greater timely accuracy.

The COVID-19-related lockdown in Denmark (from March until May 2020) represents a potential source of bias, as government restrictions on workspaces may have influenced employees’ perceptions of their work environment in 2020. However, the survey was conducted in October and November 2020, half a year after all employees were again allowed back in physical office spaces in the public sector. This diminishes a possible bias considerably. To verify this empirically, we compared descriptive statistics on employee stress, leader stress, supervisor-employee relationship, and work environment evaluations across years, and the results did not indicate bias related to the pandemic lockdown.

Moreover, while a spill-over effect of approximately 10 pct. of manager stress is no small amount, it is also clear from the analyses in this study that other variables in the work environment are important as well. The effect sizes of the control variables were slightly larger and had a higher degree of statistical certainty than the coefficient of leader stress. Stress is a complex phenomenon with many different antecedents that are most often intertwined [49]. Several work environment factors may buffer against stress. However, they are also likely to be affected by the direct leader (for example [50, 51]), and, consequently, some of the effect from leader-to-employee stress will go through such variables (as mediating effects) [49] and deflate the visible effect of leader stress. Yet, the object of this study is not to fully explain the complex phenomenon of work-related stress but rather to investigate the interwoven affective states of managers and their subordinates as well as the longevity of the effects of emotional contagion. Therefore, we have not included all possible moderating and mediating variables. However, we suggest that future studies further explore possible mediators of the relationship between leader stress and employee ditto. Furthermore, it is important to stress that organizations should focus on several organizational factors simultaneously (such as leader stress, general leadership competencies, collaborations among team-members, balancing workloads and restitution etc.) when striving to build and sustain a healthy work environment.

Finally, despite the causal robustness of the panel design employed in this study – a large N combined with variation in time –, we cannot make definitive conclusions about the direction of the stress contagion effect. It is possible that the effect is at least partially bidirectional and that the stress of employees will also spill over into the well-being of their supervisors. Unfortunately, we were not able to test such effects in this study. Still, it is theoretically likely that managers affect their employees more than vice versa: other studies in emotional contagion point to the role of social power in such transfer processes, as people of higher social status are more likely to transmit their emotional state to those of lower social status [19]. Moreover, leaders possess tools that are likely ‘gateways’ to stress contagion, such as the ability to distribute (and withhold) resources and tasks, among other things. Still, we urge future studies to investigate possible bidirectional effects of stress contagion between leaders and employees in order to address the question of causal inference further.

## Concluding remarks

This paper presents findings from a study on stress contagion effects in Esbjerg municipality in Denmark. Overall, the study contributes to corroborate the small but growing pool of empirical studies within research on management and psychosocial work environment that point to the role of managers in processes of stress ‘spill-over’ [26, 31, 33, 35]. We provide empirical evidence that managers, as highly salient group members, indeed ‘transmit’ perceived occupational stress to subordinate employees, e.g. through processes such as ‘primitive’ emotional contagion, role-modeling, and through the shaping of access to resources and controlling other important aspects of the work environment [16].

In the present paper, we bring forward new evidence of relatively persistent stress contagion effects. Even one year after managers had initially reported symptoms of occupational stress, manager stress correlates with employees’ perception of stress to a rather high degree – approximately 10 pct. of managers’ stress carries into employees’ stress. After three years, the effect is no longer traceable in our data, suggesting that stress contagion effects across hierarchical levels in organizations have an ‘expiration date’. Thus, even though the effect is traceable after the first year, it fades within two additional years after the transmission of stress symptoms.

Our study serves to emphasize the great importance of the psychosocial well-being of managers as ‘nerve centers’ for entire job teams. When managers perceive stress at their jobs, organizations run the risk of instigating a ‘domino effect’ of stress development among employees. It is therefore pivotal that organizations take on the important task of addressing the psychologically demanding sides of management within their organizations in order to tend not only to the psychosocial well-being of their common workers, but also to that of their employees in positions of power.

## Supplementary Information

**Additional file 1: Appendix I.** Descriptive statistics.

## Data Availability

Since this study contains highly sensible personal information, it is protected by the GDPR rules and follows demands on total confidentiality from the municipality. Thus, to comply with GDPR and due to the agreement with Esbjerg municipality, data access is restricted to researchers affiliated with SDU, who have signed the cooperation agreement and code of conduct statement. Hence, public access to this dataset is not possible. However, the authors encourage collaboration, and researchers with interest in using the data for scientific purposes can contact Signe Pihl-Thingvad, ssp@sam.sdu.dk.

## Footnotes

1 Public managers’ role in creating workplace social capital and its effect on employees’ well-being and health (PUMA-WSC).
