# Supplementary material for: The contagious leader: a panel study on occupational stress transfer in a large Danish municipality
Source: BMC Public Health. 2022 Oct 7;22:1874. doi: 10.1186/s12889-022-14179-5 (PMC9540037; doi:10.1186/s12889-022-14179-5)
Supplement: Supplementary file 1 — Additional file 1: Appendix I. Descriptive statistics. [file 12889_2022_14179_MOESM1_ESM.docx]

# **Appendix I. Descriptive statistics**

| **Variable** | **N** | | | **Mean** | | | **Std. dev.** | | |
| --- | --- | --- | --- | --- | --- | --- | --- | --- | --- |
|  | **2016** | **2017** | **2020** | **2016** | **2017** | **2020** | **2016** | **2017** | **2020** |
| Employee stress | 1,916 | 3,964 | 3,363 | 2.00 | 2.18 | 2.04 | .72 | .79 | .76 |
| Leader stress | 1,273 | 4,008 | 3,403 | 1.87 | 2.02 | 1.87 | .59 | .74 | .69 |
| Supervisor-employee relationship | - | 3,961 | 3,331 | - | 3.92 | 4.00 | - | .80 | .77 |
| Evaluation of work environment | 1,933 | 3,939 | 3,335 | 3.80 | 3.81 | 3.85 | .96 | 1.06 | .86 |

*Descriptives on uncentered variables reported. 2016-statistics are weighted based*
